# Supplementary figures and images for: Evaluating the Diagnostic Value of Lymphocyte Subsets in Bronchoalveolar Lavage Fluid and Peripheral Blood Across Various Diffuse Interstitial Lung Disease Subtypes
Source: Biomolecules. 2025 Jan 14;15(1):122. doi: 10.3390/biom15010122 (PMC11763757; doi:10.3390/biom15010122)

Supplementary figure S1: Gating strategy for PBMCs

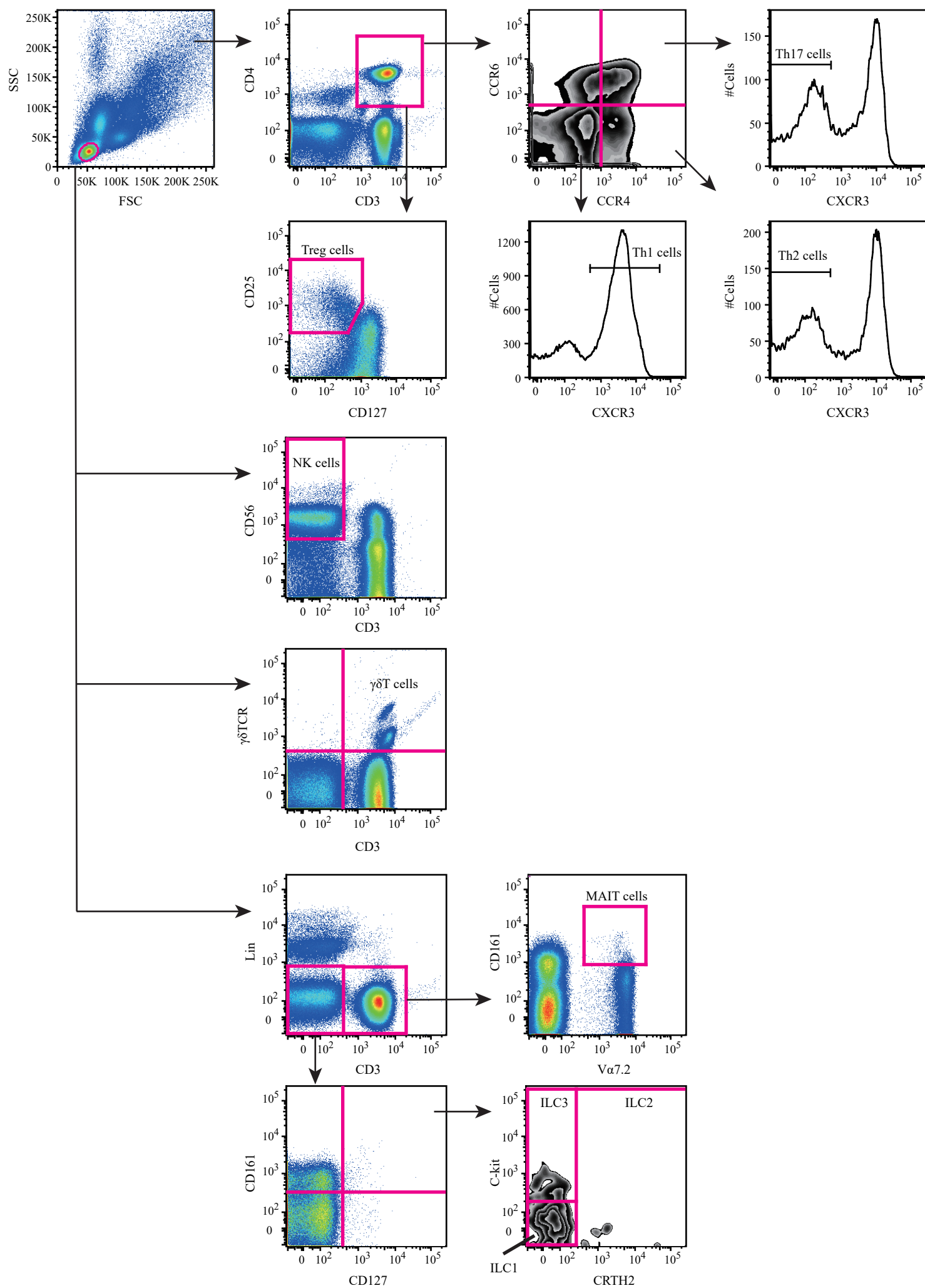

Supplement: Supplementary file 1 [file biomolecules-15-00122-s001.zip › IPBAL_Supplementary Figure S1.pdf]
